# Selenoprotein Transcript Level and Enzyme Activity as Biomarkers for Selenium Status and Selenium Requirements of Chickens (*Gallus gallus*)

**DOI:** 10.1371/journal.pone.0152392

**Published:** 2016-04-05

**Authors:** Jin-Long Li, Roger A. Sunde

**Affiliations:** 1 College of Veterinary Medicine, Northeast Agricultural University, Harbin, 150030, People’s Republic of China; 2 Department of Nutritional Sciences, University of Wisconsin, Madison, Wisconsin, United States of America; Oklahoma State University, UNITED STATES

## Abstract

The NRC selenium (Se) requirement for broiler chicks is 0.15 μg Se/g diet, based primarily on weight gain and feed intake studies reported in 1986. To determine Se requirements in today’s rapidly growing broiler chick, day-old male chicks were fed Se-deficient basal diets supplemented with graded levels of Se (0, 0.025, 0.05, 0.075, 0.1, 0.2, 0.3, 0.5, 0.75, and 1.0 μg Se/g) as Na_2_SeO_3_ (5/treatment). Diets contained 15X the vitamin E requirement, and there were no gross signs of Se-deficiency. At 29 d, Se-deficient chicks weighed 62% of Se-supplemented chicks; 0.025 μg Se/g reversed this effect, indicating a minimum Se requirement of 0.025 μg Se/g diet for growth for male broiler chicks. Enzyme activities in Se-deficient chicks for plasma GPX3, liver and gizzard GPX1, and liver and gizzard GPX4 decreased dramatically to 3, 2, 5, 10 and 5%, respectively, of Se-adequate levels, with minimum Se requirements of 0.10–0.13 μg Se/g, and with defined plateaus above these levels. Pancreas GPX1 and GPX4 activities, however, lacked defined plateaus, with breakpoints at 0.3 μg Se/g. qPCR measurement of all 24 chicken selenoprotein transcripts, plus SEPHS1, found that SEPP1 in liver, GPX3 in gizzard, and SEPP1, GPX3 and SELK in pancreas were expressed at levels comparable to housekeeping transcripts. Only 33%, 25% and 50% of selenoprotein transcripts were down-regulated significantly by Se deficiency in liver, gizzard and pancreas, respectively. No transcripts could be used as biomarkers for supernutritional Se status. For export selenoproteins SEPP1 and GPX3, tissue distribution, high expression and Se-regulation clearly indicate unique Se metabolism, which may underlie tissues targeted by Se deficiency. Based on enzyme activities in liver, gizzard, and plasma, the minimum Se requirement in today’s broiler chick is 0.15 μg Se/g diet; pancreas data indicate that the Se requirement should be raised to 0.2 μg Se/g diet to provide a margin of safety.

## Introduction

The current National Research Council (NRC) dietary selenium (Se) requirement for the broiler chicken is 0.15 μg Se/g diet [1]. This value is based primarily on studies reported in 1986 [2] which used corn-soy or semi-purified diets with analyzed dietary Se content of 0.17–0.18 μg Se/g diet; this total dietary Se level fed to day-old chicks significantly increased body weight gain and feed intake as compared to chicks fed basal diets containing 0.06–0.08 μg Se/g diet. Earlier studies showed a Se requirement of 0.1 μg Se/g diet in a crystalline amino acid diet without supplemental vitamin E, which was sufficient to prevent poor growth and exudative diathesis [3]. Furthermore, diets containing high levels of vitamin E but with <0.02 μg Se/g diet result in poor growth, poor feathering, and pancreatic atrophy [4], all of which were prevented by supplementation with 0.1 μg Se/g diet [5]. After the discovery that glutathione peroxidase (GPX) was a selenoenzyme in mammals [6], Omaye and Tappel [7] showed in 1974 that 0.12 μg total Se/g diet was the minimum level of dietary Se that would maximize plasma GPX activity in day-old chicks fed for 3 wk. A number of more recent studies have shown that Se supplementation increases tissue GPX activity in chicks, but, unfortunately, there has been a lack of studies in the chick that used multiple graded levels of supplemental Se and that used GPX activity as a biomarker for determining Se status and requirements. Just as for other nutrient requirement recommendations for poultry [8], there is a need to assess Se requirements in today’s poultry strains using biochemical and perhaps molecular biomarkers.

We have conducted an extended series of studies in the rat, using graded levels of dietary Se, to assess Se status and requirement. These studies have progress from using GPX activity [9,10], GPX protein [11], and GPX mRNA levels [12,13] as biomarkers, and we have expanded these biochemical biomarkers to include additional selenoenzyme activities [14,15] as well as using transcript levels for the full selenoproteome [16]. These studies uniformly resulted in a minimum Se requirement of 0.1 μg Se/g diet in the rapidly growing weanling male rat based on GPX1, thioredoxin reductase (TXNRD), and GPX3 activities [16]. We also found that the majority of selenoprotein transcripts are not regulated by dietary Se over the range of Se-deficient to supernutritional status (8-times the requirement), but that several selenoprotein transcripts, including GPX1, SELH and SEPW1, were highly down-regulated by Se deficiency and not up-regulated by high Se [17]. The resulting hyperbolic or sigmoidal Se response curves indicated that the minimum dietary requirements for these molecular biomarkers were less than the requirement based on biochemical biomarkers in the rat.

In the chicken, the selenoprotein genes SELV, SEPHS2, and GPX6 are not present, whereas in the avian genome SELU and SEPP2 (paralogs of Sec-containing SELV and SEPP1) are present as selenoproteins. Furthermore, while SEPP2 is not present in the mammalian genome, SEPP2 is present in the chicken genome as a Sec-containing selenoprotein [18]. We recently completed the sequencing of the turkey selenoprotein transcriptome, and confirmed this distribution in the turkey [19]. Lastly, we have determined the Se requirement for the young turkey poult using biochemical biomarkers [20], and, in parallel with the present study, we have now also assessed Se molecular biomarkers in turkey poults [21].

Thus we decided to expand this approach and evaluate biomarkers for Se status and requirements in the chicken. Our objectives were: (1) to determine the minimum dietary Se requirement in today’s rapidly growing broiler chick using Se-deficient basal diets supplemented with 10 graded levels of Se; (2) to identify Se-regulated selenoprotein transcripts (mRNAs) in the chicken, and then to determine minimum dietary Se requirements using these molecular biomarkers for Se status; (3) to evaluate the efficacy of combining individual molecular biomarkers into a panel that could be used for assessment of Se status.

## Materials and Methods

### Reagents

Molecular biology reagents were purchased from Promega (Madison, WI), Invitrogen (Carlsbad, CA), or Sigma (St. Louis, MO). All other chemicals were of molecular biology or reagent grade.

### Animals and diets

Male one-day-old chicks (n = 50, Jumbo Cornish Cross broiler, Sunnyside Hatchery, Beaver Dam, WI) were housed in battery brooder cages (5–6 per pen) with raised wire floors with 24 h lighting in the UW Poultry Research Laboratory, following the care and treatment protocol approved by the Institutional Animal Care and Use Committee at the University of Wisconsin (Protocol No. A01146). The temperature was maintained at 95°F for the first week, at 90°F for the second week, and at 85°F for subsequent weeks. Deionized water was provided in plastic waterers and diet in steel troughs, both ad libitum. The basal Se-deficient 30% torula yeast-based diet **(Table 1**) was modified from the turkey diet we used previously [20]. This diet contained 7.0% crystalline amino acids including 0.93% L-methionine, and 150 mg/kg of all-rac-alpha-tocopheryl acetate, to better match the NRC recommendations for protein and amino acids [1] and to prevent overt pancreatic atrophy. The diet provides 150% of the NRC recommendations for vitamins and minerals, except Se and vitamin E. The torula yeast is from the same lot as used in previous 30% torula yeast rodent studies where the analyzed Se content was 0.005 μg Se/g [16], and amino acids are primarily from the same lots of crystalline amino acids used for diets containing by analysis 0.003 μg Se/g diet [22]. The chicks were allocated randomly to treatment groups, and supplemented with graded levels of Se (0, 0.025, 0.05, 0.075, 0.1, 0.2, 0.3, 0.5, 0.75, and 1.0 μg Se/g) as Na_2_SeO_3_ for 29 d (5/treatment). Body weight was measured twice weekly.

**Table 1:** Basal Torula yeast-based chick diet^a^.

Ingredient	% of Diet
Torula yeast	30.00
Crystalline L-amino acids^b^	7.00
Sucrose	45.905
Lard	5.00
Mineral mix^c^	5.00
Vitamin mix^d^	0.90
Choline chloride	0.20
Vitamin E (150 mg/kg)^e^	0.015
Dicalcium phosphate	0.10
Calcium carbonate	0.70
Zn Mn Supplement^f^	0.50
Solka floc	4.68
Total:	100.00

^a^Contains 2943 kcal ME/kg diet.

^b^L-Amino acid mix (g/kg diet): Alanine, 2.75; Arginine, 7; Asparagine, 2.1; Aspartate, 2.75; Glutamate, 13.5; Glycine, 2.75; Histidine, 2.3; Isoleucine, 2.75; Leucine, 5.7; Lysine, 2.75; Methionine, 9.3; Phenylalanine, 2.75; Proline, 2.2; Serine, 2.75; Threonine, 2.75; Tryptophan, 0.95; Tyrosine, 1.65; Valine, 3.3

^c^Mineral mix (g/kg diet): CaCO_3_, 3808.35; MgCO_3_, 175; MgSO_4_-7H_2_O; 112; NaCl, 483; KCl, 756; KH_2_PO_4_, 1484; (FeNH_4_)_3_-(citrate)_4_, 143.5; KI, 0.28; MnSO_4_-1H_2_O, 23.31; NaF, 7; Al(NH_4_)(SO_4_)_2_-12H_2_O, 1.12; CuSO_4_+5H_2_O, 6.3; Na_2_MoO_4_-2H_2_O, 0.07; NiCl_2_-6H_2_O, 0.07

^d^Vitamin mix (mg/kg diet): Thiamin-HCl, 4; Riboflavin, 2.5; Pyridoxine-HCl, 2; Ca-D-pantothenate, 20; Niacin, 100; Menadione, 1; Folic acid, 2; d-biotin, 1; Vitamin B-12 (0.1% trit), 10; Retinyl palmitate (250,000 IU A/g), 0.1; Ergocalciferol (50,000 IU D/g), 0.005; Glucose monohydrate, 8757.395.

^e^DL-alpha-tocopherol acetate (Sigma T3376).

^f^Zn Mn Supplement (g/kg diet): (Zn(OH)_2_)_3_(ZnCO_3_)_2_, 0.04; MnSO_4_-1H_2_O, 0.09; sucrose, 4.87.

At day 29, chicks were killed by CO_2_ overexposure followed by exsanguination. Blood was collected in heparinized tubes, centrifuged (1500 X g, 15 min, 4°C, Eppendorf 5415R, F-45-24-11 rotor, Brinkmann, Westbury, NY) to separate plasma from red blood cells (RBC), and the RBC were reconstituted to original volume using saline phosphate buffer (76 mmol/L NaCl, 50 mmol/L sodium phosphate, pH 7.4). Liver, gizzard, and pancreas tissue samples were quickly removed, frozen on dry ice, and stored at -80°C.

### Enzyme activity assays

Tissue GPX4 activity was measured by the coupled assay procedure using 78 μM phosphatidyl choline hydroperoxide (PCOOH), the GPX4-specific substrate, as described previously [14]. Total GPX activity was assayed using 120 μM H_2_O_2_ [23], and GPX1 specific activity was calculated by subtracting the activity detected with H_2_O_2_ due to GPX4 (0.63 EU_H2O2_ /EU_PCOOH_), from the total GPX activity, as described previously [20]. Thioredoxin reductase (TXNRD) activity was assayed using 5.3 mM 5,5’-dithiobis (2-nitrobenzoic acid) (Sigma D8130), as described previously [24]. Protein concentration was determined by the method of Lowry et al [25].

### Total RNA and cDNA libraries

Total RNA was isolated from 75–100 mg of tissue homogenized in 1 ml TRIzol Reagent (cat. #15596–026, Invitrogen), following the manufacturer’s protocol, as we have done in previous studies [16]. The RNA pellet was dissolved in diethyl pyrocarbonate (DEPC)-treated water and quantitated using a ND-1000 UV-Vis Spectrophotometer (NanoDrop Technologies, Wilmington, DE). Total RNA (1 μg) was reverse transcribed to cDNA using the RETROscript kit (AM1710, Ambion Inc., Austin, TX), following the manufacturer’s protocol and using the Ambion Oligo(dT) primer. Working stocks of cDNA libraries were diluted 1/50 in DEPC-treated water.

qPCR

Quantitative reverse transcription polymerase chain reaction (qPCR) primers were designed using Primer3 or Primer3Plus (http://primer3plus.com/cgi-bin/dev/primer3plus.cgi). Gene-specific primers were typically designed as 20-mers with a dissociation temperature of ~60°C, to span apparent splice-junctions and amplify 120–150 basepair (bp) fragments based on NCBI *Gallus gallus* nucleotide annotation (**Table 2**). qPCR primers were screened by PCR reactions against reverse-transcribed cDNA working stocks followed by gel electrophoresis to confirm expected fragment size. Lastly, preliminary qPCR reactions were conducted to verify that the product yielded a single dissociation (derivative) peak and consistent amplification signal.

**Table 2:** qPCR primers for chicken selenoproteinsa.

Gene	Forward Sequence	Reverse Sequence	Fragment^b^
			(bp)
GPX1	GCGACTTCCTGCAGCTCAACGA	CGTTCTCCTGGTGCCCGAAT	99
GPX2	CGCCAAGTCCTTCTACGACCT	CCTCAGAGCGACGCCACGTT	111
GPX3	ATCCCCTTCCGAAAGTACGC	GACGACAAGTCCATAGGGCC	129
GPX4	CGGTGAATTACACTCAGCTCGT	CTTTGATCTGCGCGTCGTCC	123
DIO1	AAGCTGCACCTGACCTTCATT	TTGTTTCTGAAGGCCCATCCA	138
DIO2	CAGTGTAATCCACATAGCCA	CTGAGCCAAAATTAACCACC	137
DIO3	GACACCATGGACAACGCTTC	GCCCTGGTACATCACCTTCTC	90
EPT1	GCTGGCCCCAAATCTCATAAC	CCACAACGACCCATACTCCAT	140
MSRB1	CCGCGCCAAATACGAGCACT	CAGCCCATTGCCACACTTGCC	130
SELH	CCCTGGCCGTAGAGATCAACC	GCTCGGGGAACTTCAGCTTG	138
SELK	CTGAGGAGAAGAGGCTACACATC	GATTTATTCTGCCCATTCTACGG	97
SELM	CTTCGTCAGCCAGGACATCCC	GCTCCTCGTATCTGAAGCTAAGCA	101
SELO	GCTCAGAATGCCATAGAAGC	TCTTTTGTCTCCATCTCCGTG	119
SELT	TCATAGCCCCATCTATCAGCAC	AACGTGACTGCAAGAGAAGCATCC	139
SELU	CTTTCAGGCTTCTTCCGCATT	TGCTCCAATCACATACACTCC	120
SEPN1	TTTACGGGTTACATCGTCCT	ACCTATATCCACCTCCATGTTGC	144
SEPP1	CTAGCTGATACTTGTGCCTC	CACGTATGAGATGTTGACCAG	92
SEPP2	AAGGACTTCTGCGGGAACTGCTC	TTCTCCTCCTGTTTGGGAAGCG	107
SEP15	TGACAAGCCTAAGCTCT	CAATGTTCCCACTGTCGTC	102
SEPW1	CTCCGCGTCACCGTGCTCT	CTGCCCACCGTCACCTCGAAC	155
TXNRD1	ACTGGATGACTATGACCGAA	TATGCATTCTCATACGTGAC	103
TXNRD2	CTACACATATTACGGGCGCACT	ACATAGCTGGCTCCAACAACC	103
TXNRD3	CCTGGCAAAACGCTAGTTGT	TCTCTTGGTCAAAGCCTCGAA	127
VIMP	CACCTCATCAGCAGTCCCGAA	TAGCCTCATCCACCCGCAGA	141
SEPHS1	GCTGCTGGACTTATGCACACT	AGGACACCTCATTTCGCTGCT	112
GAPDH	AATGAGAGGTTCAGGTGCCC	ACCAGACAGCACTGTGTTGG	150
ACTB	ACACACGGACACTTCAAGGG	TACTCAGCACCTGCATCTGC	128

^a^Primers used for qPCR based on NCBI transcript sequences. Sequences are written 5’ to 3’.

^b^Resulting PCR fragment as predicted by transcript sequences and verified by PCR followed by gel electrophoresis.

qPCR analyses were conducted using 96-well plates. For full Se response curves, individual cDNAs (n = 3-4/treatment were analyzed in triplicate where the final 25 μL reactions contained 10 ng reverse transcribed RNA (10 μl of 1/50 working stock), 0.2 mmol/L gene specific forward and reverse primers, and 1X SybrGreen PCR Master Mix (#4309155, Applied Biosystems, Foster City, CA), according to manufacturer’s protocols. Reactions were followed in an ABI Prism 7000 (Applied Biosystems) with initial stages of 50°C for 2 min and 95°C for 10 min, followed by 50 cycles consisting of 95°C for 15 sec and 60°C for 2 min. A dissociation curve was run for each plate to confirm the production of a single product. The amplification efficiency for each gene was determined using the DART-PCR program [26]. The mRNA relative abundance was calculated according to Pfaffl [27], accounting for gene-specific efficiencies, and normalized to the mean of β-Actin (ACTB) and glyceraldehyde-3-phosphate dehydrogenase (GAPDH), and expressed as a percentage of the plateau level in the resulting Se response curve. Initial screening for relative transcript expression was conducted on individual samples from chicks fed the Se-deficient, Se-adequate, and high-Se diets (0, 0.3 and 1.0 μg Se/g diet, respectively, n = 4/treatment) for liver and gizzard; for pancreas, samples from the 0.025 μg Se/g diet group were also analyzed. To compare transcript expression of different selenoproteins, relative abundance was normalized for basepair length of the amplified fragment [19].

### Analysis

Data are presented as mean±SEM. All data were analyzed by ANOVA to test for the main effect of diet. Growth rates (weight gain/day) were compared using ANCOVA. When the main effect of diet was significant, differences between means were assessed by Duncan’s multiple range analysis (*P*<0.05), with Kramer’s modification for unequal class sizes where necessary [28]. When variance equality was significant, as tested by modified Levene’s median test (*P*<0.05), significant differences between means were assessed instead by Scheffé’s F-test. For each biomarker, a “Se response curve” was constructed using sigmoidal or hyperbolic regression analysis (Sigma Plot, Jandel Scientific) on all individual values at each dietary Se level as described previously [12,13,29]; the “plateau breakpoint” for each Se response curve, defined as the intersection of the line tangent to the point of steepest slope and the plateau, was calculated to estimate the minimum dietary Se necessary to obtain the plateau response.

## Results

### Growth

**Fig 1:**
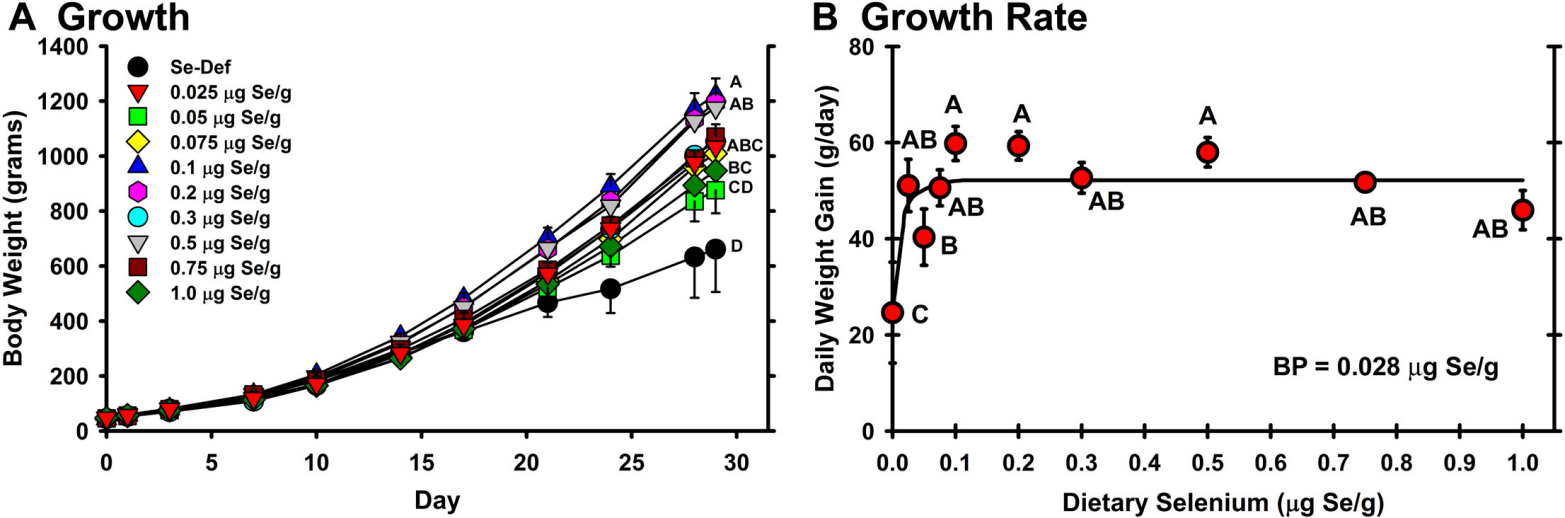
Effect of dietary Se on growth and growth rate in chicks. A. Body weights of day-old male chicks supplemented with graded levels of dietary Se at the indicated levels for 29 d (n = 5/treatment) and weighed biweekly. Values are the mean weight±SEM. Means at day 29 without a common letter are significantly different (*P*<0.05). B. Daily weight gain of chicks over days 14–29, calculated as g/day. Hyperbolic line is the resulting Se response curve with a breakpoint (BP) of 0.028 μg Se/g diet, calculated as described in the text. Means without a common letter are significantly different (*P*<0.05).

The chicks were allocated randomly to dietary treatment. At the start, the average weight was 45.7±0.6 g, and there were no significant differences (*P* = 0.78) in initial group weights (**Fig 1A**). By day 17, dietary Se had a significant effect on body weight (*P* = 0.002) and chicks fed the Se-deficient diet had the lowest average body weight. This effect persisted such that Se-deficient chicks weighed 661±158 g whereas the chicks in the other groups averaged 1065±25 g. The Se response curve for the day 29 body weight yielded a plateau breakpoint of 0.026 μg Se/g diet (**Table 3**). Similarly, the growth rate of the Se-deficient group over the last 15 days of the study was half of the growth rate of the other groups (*P*<0.05) (**Fig 1B**), with a plateau breakpoint of 0.028 μg Se/g diet.

**Table 3:** Se requirement hierarchy in growing chicks.

Biomarker		Extent of Regulation		Minimum Requirement
**Growth**		**%**^a^	**P-value**	**(μg Se/g diet)**
	Final Weight	62.8	0.0003	0.026
	Daily Gain (14–29 d)	46.8	<0.05	0.028
**Enzyme Activity**		**%**^a^	**P-value**	**(μg Se/g diet)**
	Liver TXNRD Activity	32.9	0.0001	0.10
	Liver GPX4 Activity	9.7	4.2E-10	0.10
	Plasma GPX3 Activity	2.6	4.8E-09	0.11
	Gizzard GPX1 Activity	5.1	2.3E-08	0.13
	Liver GPX1 Activity	1.8	2.9E-19	0.13
	Gizzard GPX4 Activity	10.8	2.9E-17	0.13
	RBC GPX4 Activity	9.4 ^b^	4.6E-26	0.30
	Pancreas GPX1 Activity	38.9 ^b^	0.0117	0.31
	Pancreas GPX4 Activity	25.0 ^b^	5.5E-05	0.34
**Liver Transcript Levels**		**%**^a^	**P-value**	**(μg Se/g diet)**
	Liver SELM mRNA	43.9	0.0373	0.04
	Liver GPX4 mRNA	35.5	0.0001	0.07
	Liver SELH mRNA	15.8	3.1E-06	0.07
	Liver DIO1 mRNA	45.1	0.0365	0.08
	Liver SEPP1 mRNA	44.7	0.0131	0.09
	Liver SELU mRNA	27.6	0.0240	0.10
	Liver GPX3 mRNA	25.7	0.0128	0.10
	Liver GPX1 mRNA	39.4	0.0007	0.11
**Gizzard Transcript Levels**		**%**^a^	**P-value**	**(μg Se/g diet)**
	Gizzard SELH mRNA	33.9	0.0006	0.03
	Gizzard GPX4 mRNA	55.6	0.0001	0.05
	Gizzard SELU mRNA	51.0	0.0173	0.05
	Gizzard SELM mRNA	68.4	0.0242	0.05
	Gizzard GPX1 mRNA	56.4	0.0453	0.06
	Gizzard GPX3 mRNA	48.4	0.0118	0.08
**Pancreas Transcript Levels**		**%**^a^	**P-value**	**(μg Se/g diet)**
	Pancreas MSRB1 mRNA	29.7	0.0036	0.02
	Pancreas SELK mRNA	19.7	1.7E-07	0.04
	Pancreas SEPP1 mRNA	32.3 ^b^	0.0050	0.04
	Pancreas SEP15 mRNA	21.8 ^b^	0.0224	0.05
	Pancreas GPX1 mRNA	44.5 ^b^	0.0247	0.07
	Pancreas VIMP mRNA	18.0	0.0002	0.08
	Pancreas SEPW1 mRNA	52.4	0.0034	0.09
	Pancreas SELM mRNA	31.8 ^b^	0.0251	0.14
	Pancreas SELU mRNA	17.8 ^b^	4.4E-06	0.15
	Pancreas GPX3 mRNA	37.5	0.0232	0.16
	Pancreas GPX4 mRNA	27.1	0.0001	0.18
	Pancreas SELH mRNA	14.3 ^b^	2.7E-06	0.21
**Panel Transcript Levels**		**%**^a^	**P-value**	**(μg Se/g diet)**
	Gizzard	52.2	0.0007	0.03
	Liver	34.7	0.0001	0.08
	Pancreas	27.3^b^	2.5E-05	0.13

^a^Extent of Regulation: percentage of Se-deficient as compared to Se-adequate plateau

^b^Extent of Regulation: percentage of Se-deficient as compared to 0.3 μg Se/g diet

### General health

In these chicks supplemented with 15-times the NRC vitamin E requirement [1] and adequate sulfur amino acids, there were no gross signs of Se deficiency. Weight of the pancreas has been reported to be decreased in Se deficiency [4], but pancreas weight as well as liver and gizzard weights in our study averaged 0.23, 3.08, and 1.07%, respectively, when expressed as percent body weight, and were not significantly altered by dietary Se (data not shown).

### Enzyme Activity Analyses

**Fig 2:**
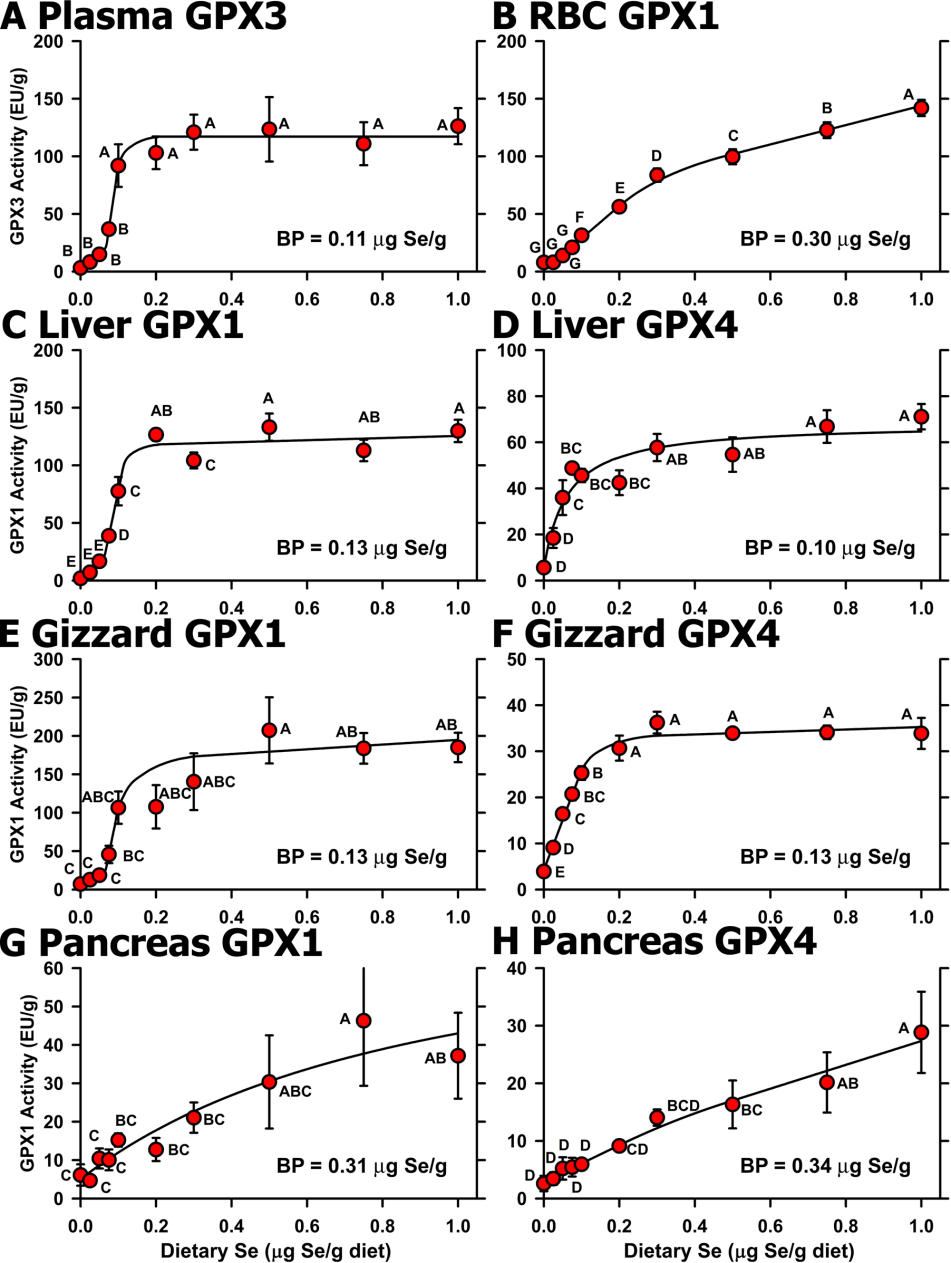
Effect of dietary Se on selenoenzyme activity. Activities for plasma GPX3 (A), RBC GPX1 (B), liver GPX1 (C), liver GPX4 (D), gizzard GPX1 (E), gizzard GPX4 (F), pancreas GPX1 (G), and pancreas GPX4 (H) in chicks supplemented with the indicated graded levels of dietary Se for 29 d. Activities are expressed as enzyme unit (EU)/g protein. Values are the mean±SEM (5/treatment). Means without a common letter are significantly different (*P*<0.05). Overall level of significance, as determined by ANOVA, is given in Table 3. Se response curve breakpoints (BP) are indicated in each panel, calculated as described in the text.

The Se response curves for selenoenzyme activity clearly show that the basal diet was Se deficient. Plasma GPX3 activity in Se-deficient chicks was 3% of Se-adequate levels (**Fig 2A**). Graded dietary Se supplementation resulted in a sigmoidal Se response curve for plasma GPX3 activity with a plateau breakpoint at 0.11 μg Se/g diet (**Table 3**). Super-nutritional levels of Se supplementation (0.5 μg Se/g diet and higher) did not result in further increases in GPX3 activity.

RBC GPX1 activity in Se-deficient chicks decreased to 9% of levels in Se-adequate chicks (0.3 μg Se/g diet) (**Fig 2B**). With increasing dietary Se, RBC GPX1 activity increased and reached a breakpoint at 0.3 μg Se/g diet. As observed in young rats supplemented with graded levels of Se [9,12,13,14], RBC GPX1 activity continued to increase with higher levels of dietary Se but at a rate 50% of the rate before 0.3 μg Se/g diet.

Liver and gizzard GPX1 activities in Se-deficient chicks were 2% and 5%, respectively, of Se-adequate animals (**Fig 2C and 2E**). As with plasma GPX3 activity, GPX1 activity in liver and gizzard increased sigmoidally with increasing dietary Se, reaching defined plateaus with breakpoints at 0.13 μg Se/g diet.

Liver and gizzard GPX4 activities in Se-deficient chicks were 10% and 5%, respectively, of Se-adequate animals. In contrast to GPX1, however, liver and gizzard GPX4 activity increased hyperbolically with increasing supplemental Se, reaching plateau breakpoints of 0.10 and 0.13 μg Se/g diet, respectively (**Fig 2D and 2F**).

In pancreas, the GPX1 and GPX4 activity response to increasing dietary Se was distinct from that observed in liver and gizzard (**Fig 2G and 2H**). Pancreas GPX1 and GPX4 activities in Se-deficient chicks decreased to 39 and 25%, respectively, of levels in Se-adequate chicks (0.3 μg Se/g diet), with breakpoints at 0.31 and 0.34 μg Se/g diet, respectively, but then continued to increase beyond 0.3 μg Se/g diet. Pancreas GPX1 activity in chicks fed 1.0 μg Se/g diet was significantly different from levels in chicks fed 0.1 μg Se/g diet, and pancreas GPX4 activity in chicks fed 1.0 μg Se/g diet was significantly different from levels in chicks fed 0.3 μg Se/g diet.

Liver TXNRD activity was mildly but significantly reduced in Se-deficient chicks to 33% of Se-adequate plateau levels (**Table 3**). Graded Se supplementation resulted in a well-defined plateau with a plateau breakpoint at 0.10 μg Se/g diet (data not shown).

### Selenoprotein mRNA Analyses

We previously used microarray analysis to study the selenoproteome of the rat and found that the majority of selenoprotein transcripts were not regulated by Se status, but that GPX1, SELH and SEPW1 transcripts were highly down-regulated by Se deficiency [16]. Furthermore, we found that these transcripts could be used as molecular biomarkers of Se status.

**Fig 3:**
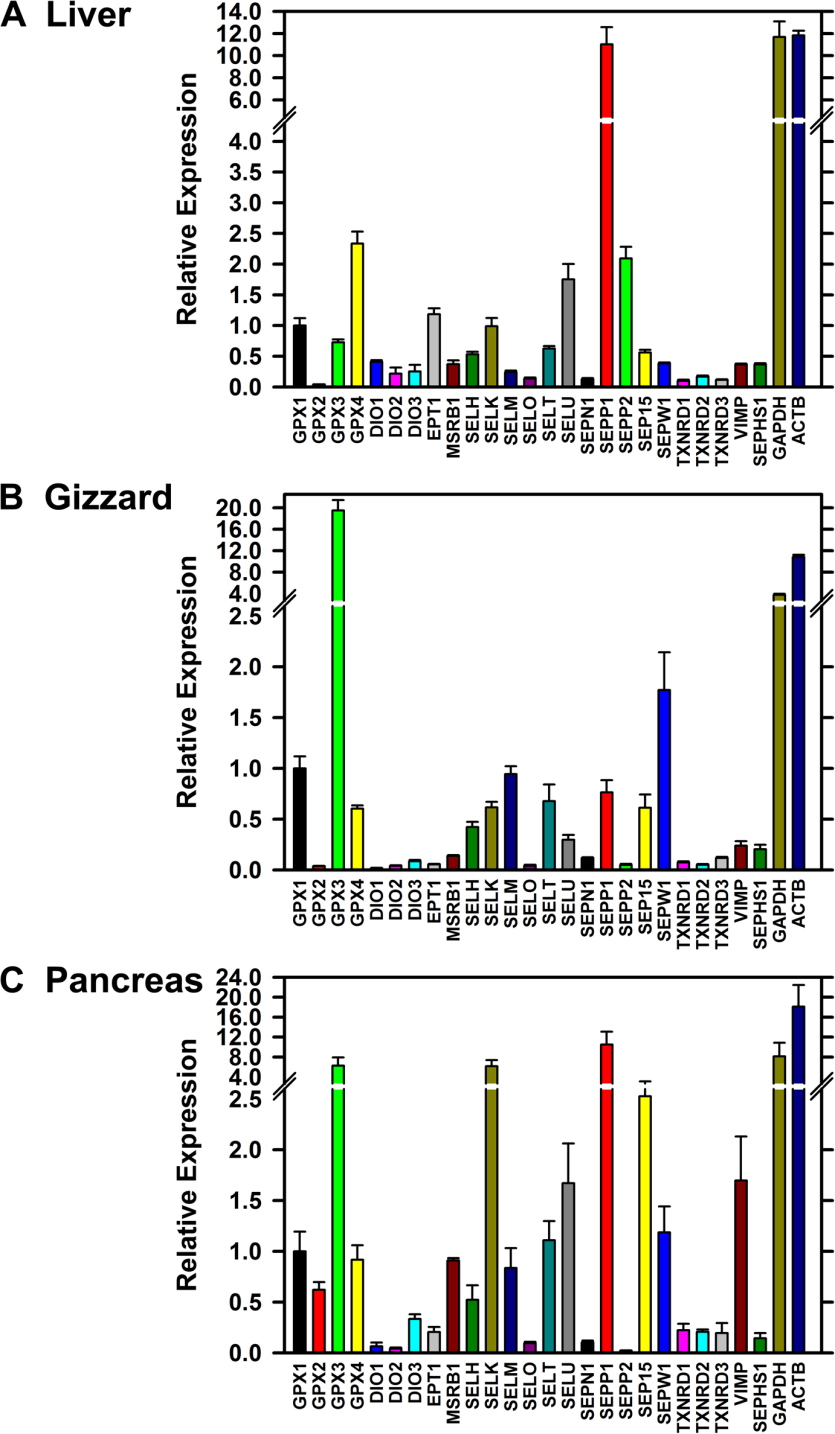
**Relative expression of selenoprotein transcripts in Se-adequate chick liver (A), gizzard (B) and pancreas (C).** Relative transcript expression for each gene was determined by qPCR and expressed relative to the level of GPX1 transcript, as described in the text. Se-adequate tissues were from chicks fed 0.3 mg Se/g diet. Primer pairs used for these analyses are listed in Table 2. Bars show the mean±SEM (n = 4).

We initially screened reverse-transcribed liver, gizzard, and pancreas cDNA libraries for selenoprotein transcript expression using qPCR analysis of cDNA libraries from 4 chicks in each treatment (0, 0.3 and 1.0 μg Se/g diet for liver and gizzard, and for 0, 0.025, 0.3 and 1.0 μg Se/g diet for pancreas). **Fig 3** shows the relative expression of transcripts for the 24 chicken selenoproteins, for SEPHS1, and for the housekeeping genes ACTB and GAPDH. In liver (**Fig 3A**), SEPP1 mRNA was the most abundant and expressed at levels comparable to levels for GAPDH and ACTB. Also highly expressed was mRNA for GPX4, expected due to the high levels of liver GPX4 activity, but also for SELU and SEPP2, two selenoproteins not found in mammals. In gizzard (**Fig 3B**), GPX3 was the most abundant transcript, expressed at levels higher than for GAPDH and ACTB and 19-fold higher than for GPX1. SEPW1 mRNA was also highly expressed. In pancreas (**Fig 3C**), SEPP1, GPX3, and SELK mRNA were highly expressed and at levels comparable to GAPDH and ACTB.

**Fig 4:**
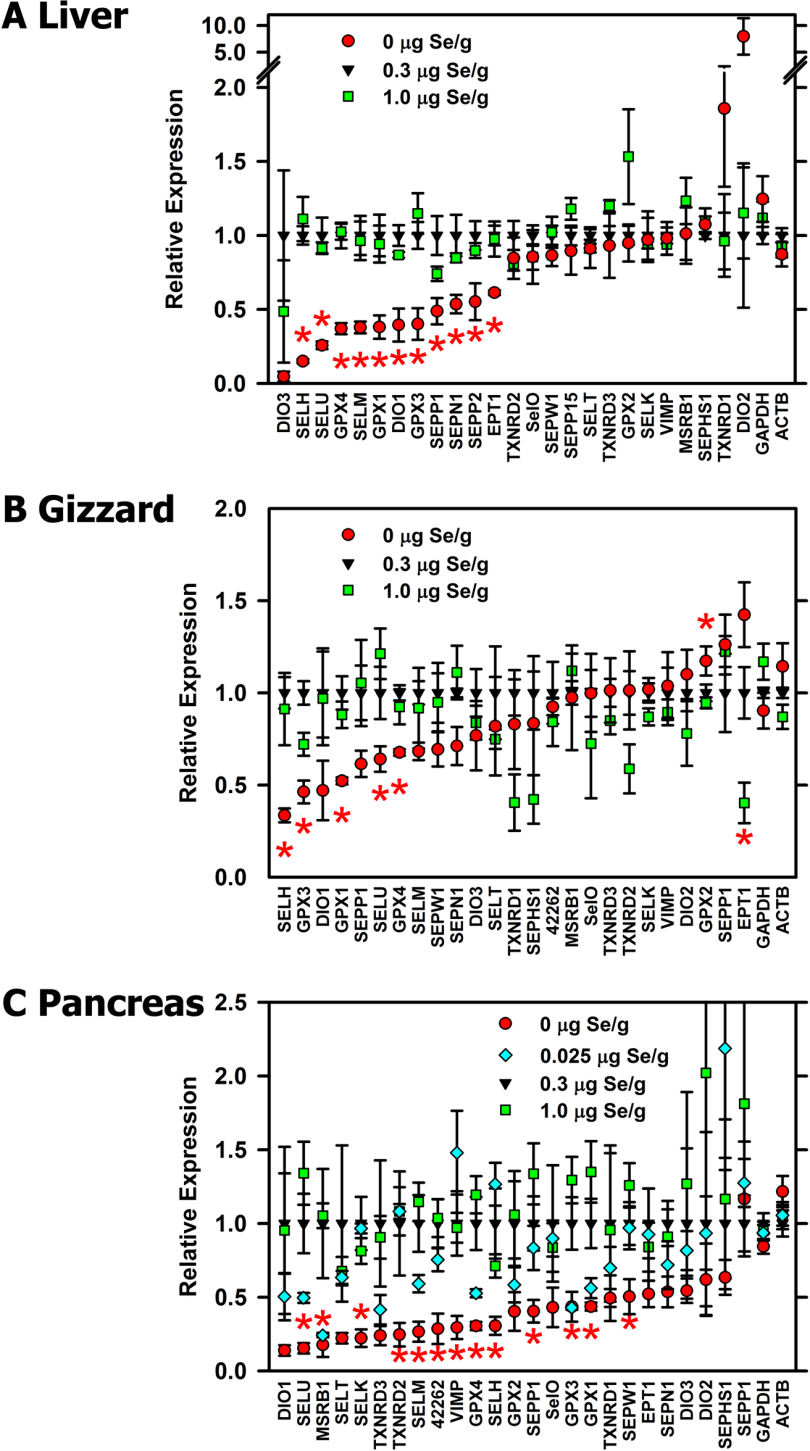
**Effect of Se status on relative expression of selenoprotein transcripts in chick liver (A), gizzard (B) and pancreas (C).** Liver and gizzard RNA was from chicks supplemented with 0, 0.3, or 1.0 μg Se/g diet. Pancreas RNA was from chicks supplemented with 0, 0.025, 0.3, or 1.0 μg Se/g diet. Relative transcript expression for each gene was determined by qPCR and expressed relative to the level in Se-adequate (0.3 μg Se/g diet) Bars show the mean±SEM (n = 4). Asterisks indicated significant effects of dietary treatment (*P*<0.05).

Our initial screen also allowed us to identify transcripts significantly regulated by Se deficiency or high-Se status (**Fig 4**). Neither transcript expression of GAPDH nor ACTB was significantly regulated by Se status in any tissue. Similarly, 1.0 μg Se/g diet did not significantly increase expression of any transcript; apparent but non-significant increases were only observed for poorly-expressed transcripts. In liver (**Fig 4A**), 11 selenoprotein transcripts were significantly down-regulated by Se deficiency in this initial screen. In gizzard (**Fig 4B**), 5 selenoprotein transcripts were significantly down-regulated by Se deficiency in this initial screen; GPX2 mRNA was significantly increased by Se deficiency, and EPT1 mRNA increased by high Se in this initial screen but not in the complete screens (see below). In pancreas (**Fig 4C**), 13 selenoprotein transcripts were significantly down-regulated by Se deficiency, and 6 of these transcripts remained significantly down-regulated by in chicks fed 0.025 μg Se/g diet in this initial screen

**Fig 5:**
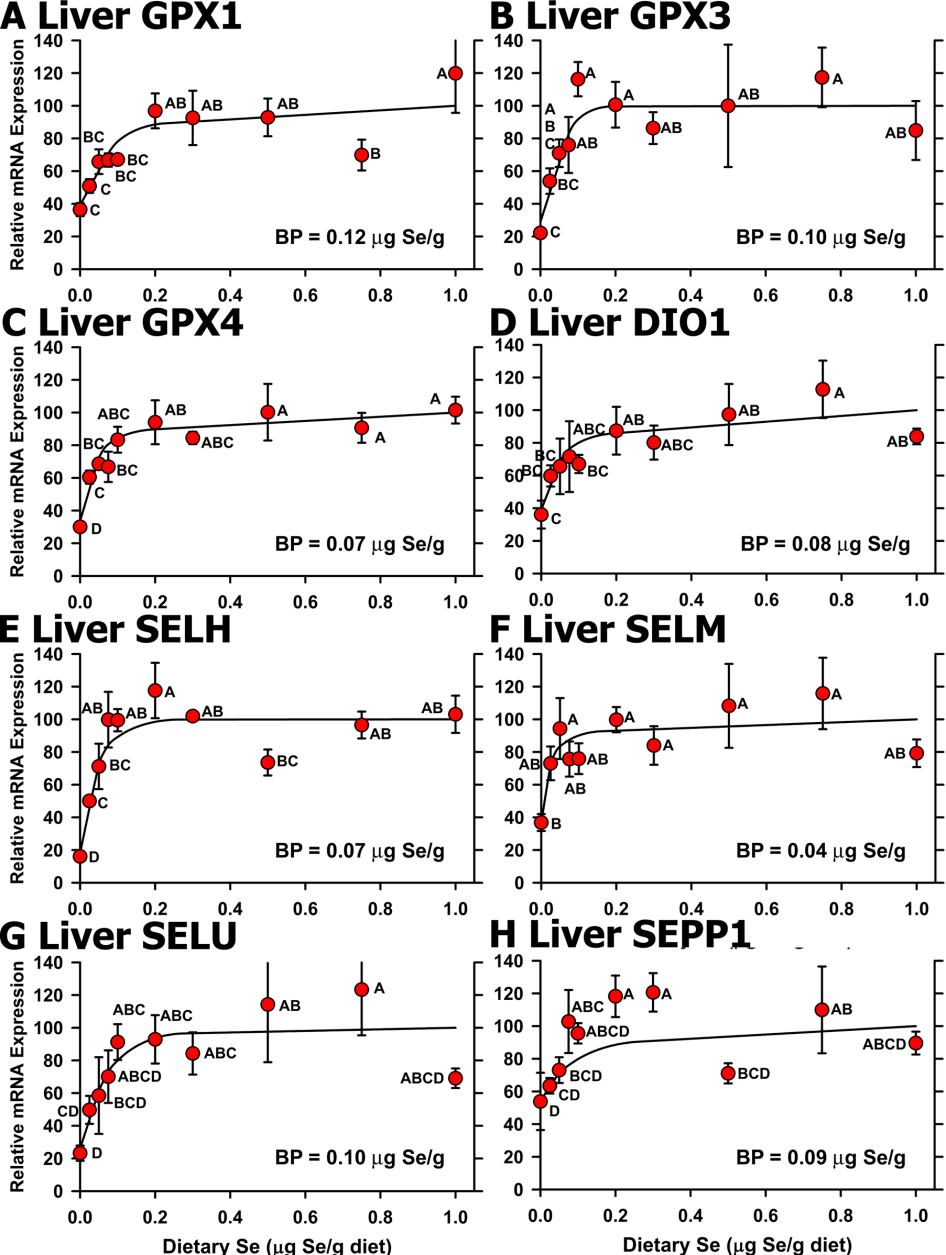
Effect of dietary Se on significantly-regulated selenoprotein transcript level in chick liver. Relative transcript levels are plotted for GPX1 (A), GPX3 (B), GPX4 (C), DIO1 (D), SELH (E), SELM (F), SELU (G) and SEPP1 (H) in chicks supplemented with the indicated levels of dietary Se for 29 d. Values were determined in triplicate for each sample, normalized to the mean of GAPDH and ACTB levels in each sample, expressed as a percentage of Se-adequate plateau levels, and plotted as mean±SEM (n = 4/treatment). Means without a common letter are significantly different (*P*<0.05). Overall level of significance, as determined by ANOVA, is given in Table 3. Se response curve breakpoints (BP) are indicated in each panel, calculated as described in the text.

**Fig 6:**
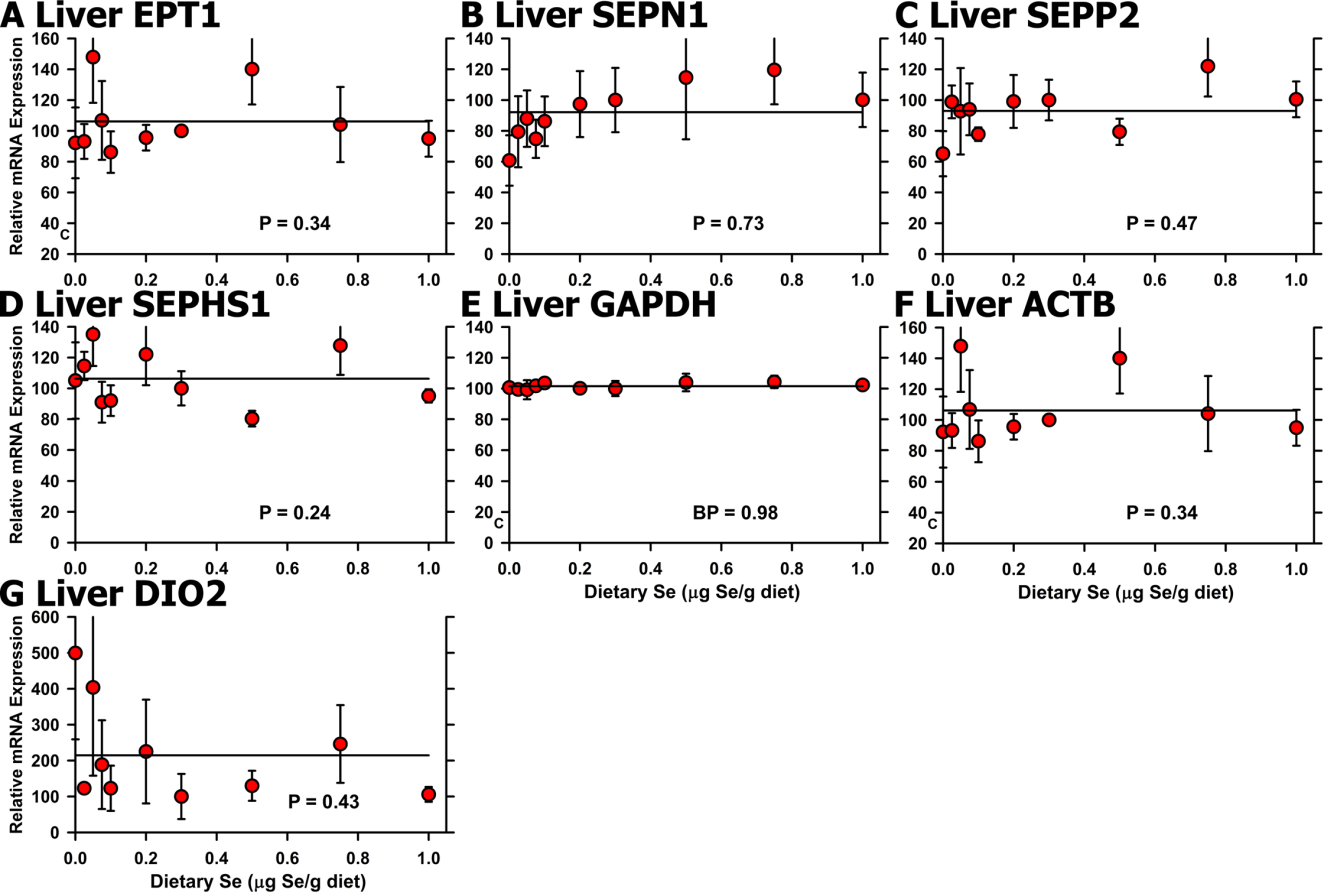
Effect of dietary Se on unregulated selenoprotein transcript levels in chick liver. Relative transcript levels are plotted for EPT1 (A), SEPN1 (B), SEPP2 (C), SEPHS1 (D), GAPDH (E), ACTB (F), and DIO2 (G) in chicks supplemented with the indicated levels of dietary Se for 29 d. Values were determined in triplicate for each sample, normalized to the mean of GAPDH and ACTB levels in each sample, expressed as a percentage of Se-adequate plateau levels, and plotted as mean±SEM (n = 3-4/treatment). Overall level of significance, as determined by ANOVA, is indicated in each panel.

We next conducted qPCR analyses on cDNA libraries from all 10 dietary Se levels (n = 3 or 4 for each treatment) for selenoprotein transcripts that were significantly regulated in the initial screen. In liver, Se regulation based on all 10 treatments remained significant for 8 selenoproteins, or 33% of the selenoproteome (**Fig 5**). In all cases, the resulting Se response curves showed clearly-defined plateaus, showing that high-Se status (1.0 μg Se/g diet) did not increase selenoprotein mRNA levels in liver. Se deficiency decreased mRNA levels significantly, ranging from 16% (SELH) of Se-adequate levels to 45% (SELM, DIO1), with plateau breakpoints in a narrow range from 0.04–0.11 μg Se/g diet. Analysis based on all 10 levels of dietary Se, failed to show significant Se regulation for liver EPT1, SEPN1 and SEPP2 (*P* = 0.34 to 0.73) (**FIG 6A, 6B and 6C**), which had been identified in the initial screen for Se regulation, showing that these transcripts could not be used as molecular biomarkers of Se status. Liver SEPHS1 as well as GAPDH and ACTB mRNA were also not regulated significantly (**Fig 6D, 6E and 6F**). Liver DIO2 mRNA, which was non-significantly up-regulated in Se deficiency in the initial screen, remained non-significantly up-regulated when we examined the full Se response curve for liver DIO2 mRNA (**Fig 6G**).

**Fig 7:**
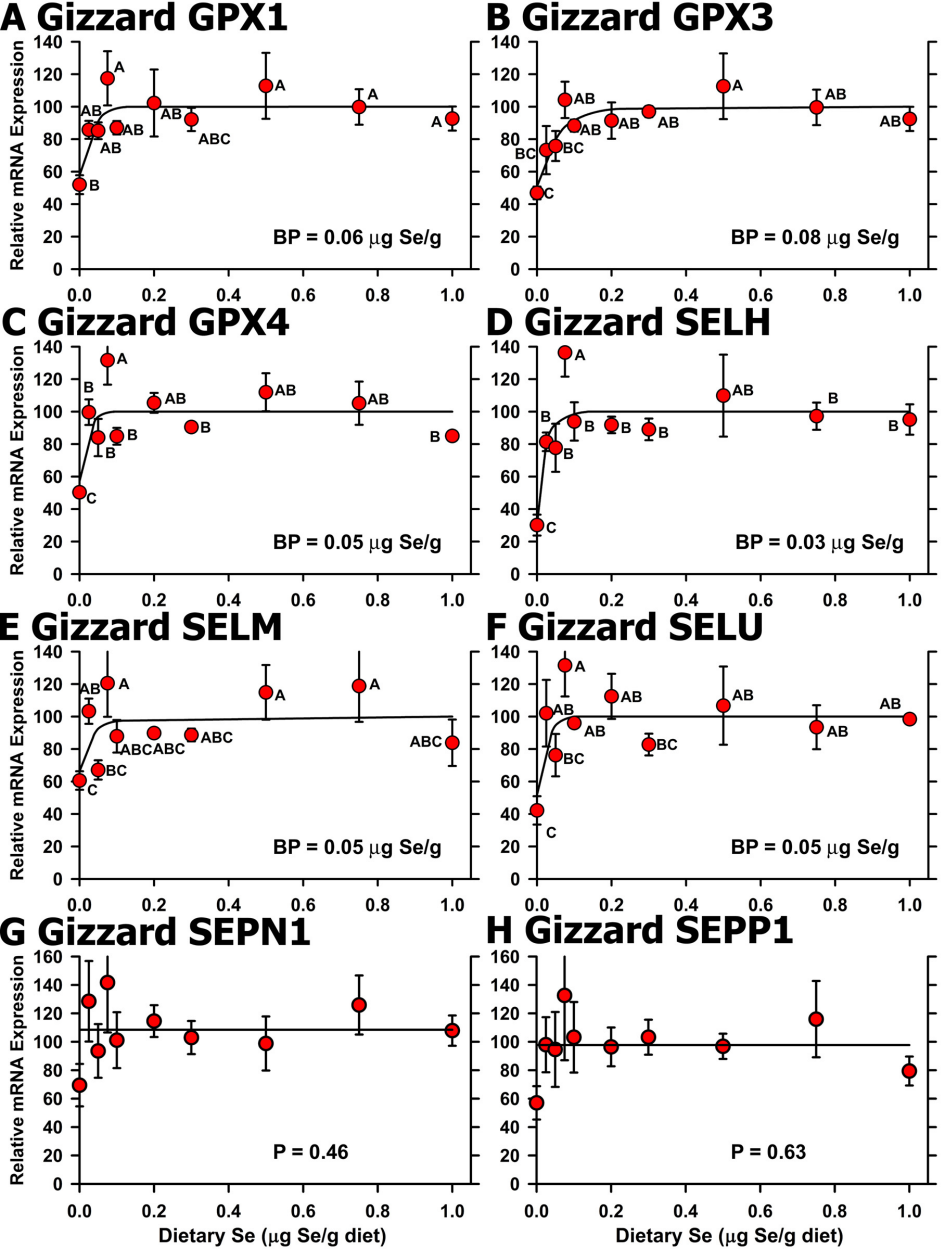
Effect of dietary Se on selenoprotein transcript level in chick gizzard. Relative transcript levels are plotted for GPX1 (A), GPX3 (B), GPX4 (C), SELH (D), SELM (E), SELU (F), SEPN1 (G), and SEPP1 (H) in chicks supplemented with the indicated levels of dietary Se for 29 d. Values were determined in triplicate for each sample, normalized to the mean of GAPDH and ACTB levels in each sample, expressed as a percentage of Se-adequate plateau levels, and plotted as mean±SEM (n = 3-4/treatment). For panels A-F, means without a common letter are significantly different (*P*<0.05). Overall level of significance, as determined by ANOVA, is given in Table 3. Se response curve breakpoints (BP) are indicated in each panel, calculated as described in the text. For panels G and H, overall level of significance, as determined by ANOVA.

In gizzard, qPCR analyses for Se regulation based on all 10 treatments remained significant for 5 of the 6 regulated selenoprotein transcripts identified in the initial screen, plus SELM (**Fig 7A–7F)**. As in liver, high-Se status (1.0 μg Se/g diet) did not increase selenoprotein mRNA levels. Se deficiency decreased mRNA levels significantly for SELH, GPX3, SELU, GPX4, GPX1, and SELM, ranging from 34% (SELH) of Se-adequate levels to 68% (SELM), with plateau breakpoints in a narrow range from 0.03–0.08 μg Se/g diet. EPT1 mRNA, which was identified as up-regulated in the initial screen for Se regulation, was not significantly regulated (*P* = 0.91) (data not shown), as were SEPN1 and SEPP1 mRNA levels (**Fig 7G and 7H**).

**Fig 8:**
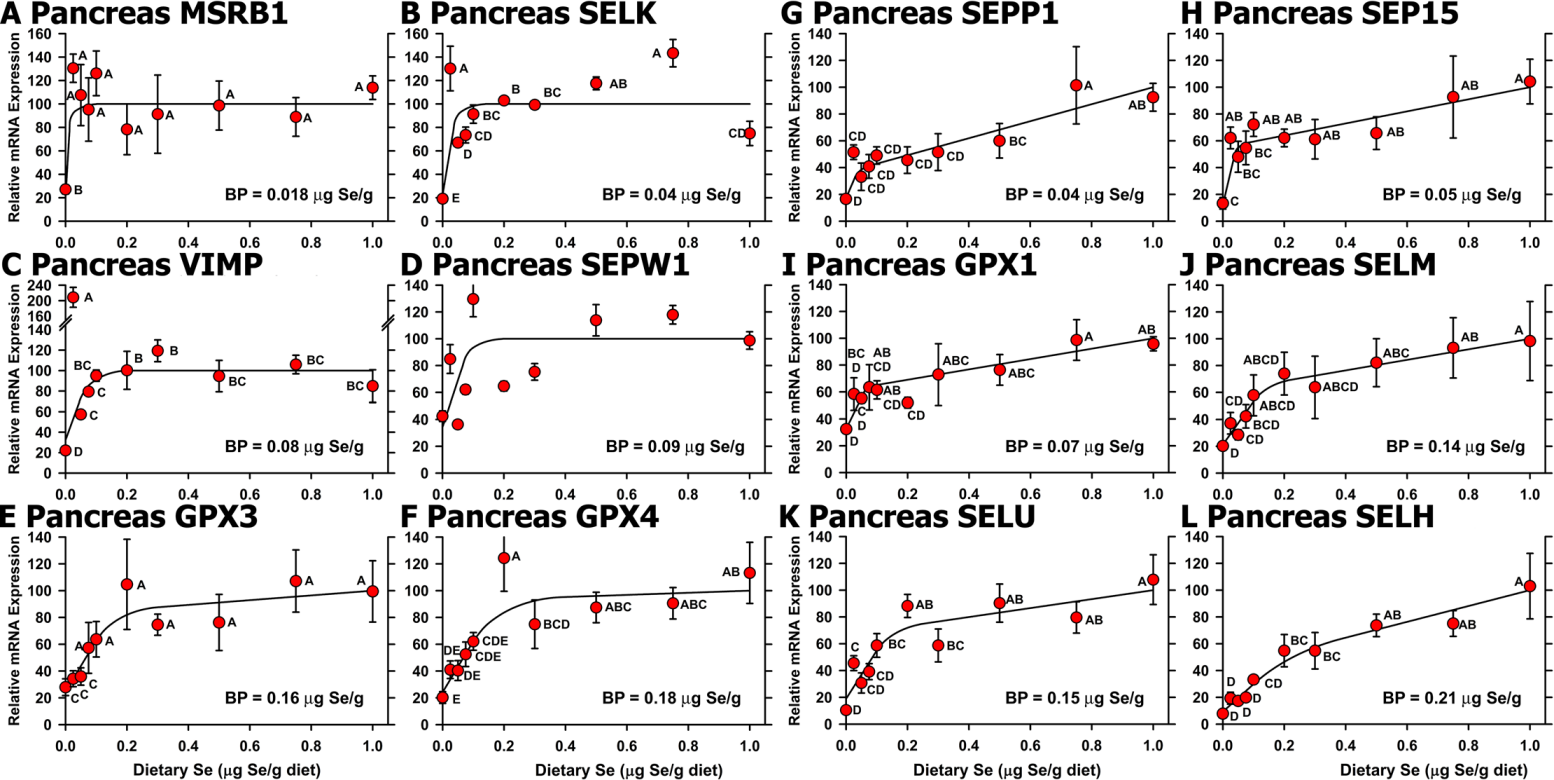
Effect of dietary Se on significantly-regulated selenoprotein transcript level in chick pancreas. Relative transcript levels are plotted for MSRB1 (A), SELK (B), VIMP (C), SEPW1 (D), GPX3 (E), GPX4 (F), SEPP1 (G), SEP15 (H), GPX1 (I), SELM (J), SELU (K), and SELH (H) in chicks supplemented with the indicated levels of dietary Se for 29 d. Values were determined in triplicate for each sample, normalized to the mean of GAPDH and ACTB levels in each sample, expressed as a percentage of Se-adequate plateau levels, and plotted as mean±SEM (n = 3-4/treatment). Means without a common letter are significantly different (*P*<0.05). Overall level of significance, as determined by ANOVA, is given in Table 3. Se response curve breakpoints (BP) are indicated in each panel, calculated as described in the text.

In pancreas, Se regulation based on all 10 treatments remained significant for 12 of the 13 selenoprotein transcripts identified in the initial screen (**Fig 8A–8L)**. These Se-regulated transcripts included GPX3, SELK, SEPP1, and SEP15, which were the transcripts highly-expressed in pancreas (**Fig 3C**). Well defined Se-adequate plateaus in the Se response curves were observed for 6 of these transcripts (**Fig 8A–8F)**, but transcript levels for SELM, SELU, GPX1 and SEP15 continued to increase non-significantly from 0.2 to 1.0 μg Se/g diet at (**Fig 8H–8K**). Se deficiency decreased mRNA levels significantly in pancreas for SELH, SELU, VIMP, and SELK to less than 20% of levels in chicks fed 0.3 μg Se/g diet. SEPP1 mRNA (**Fig 8G)**, which was expressed at 8-times the level of GPX1 in Se-adequate pancreas, fell to 32% of 0.3 μg Se/g diet levels, increased linearly with increasing supplementation up to 0.1 μg Se/g diet, but then continued to increase at half the rate from 0.2 to 1.0 μg Se/g diet, so that SEPP1 mRNA levels in Se-deficiency were 18% of levels at 1.0 μg Se/g diet. SELH mRNA ((**Fig 8L)**, which was expressed at half the level of GPX1 in Se-adequate pancreas, fell to <20% of 0.3 μg Se/g diet levels, increased linearly with increasing supplementation up to 0.2 μg Se/g diet, but then continued to increase at half the rate from 0.2 to 1.0 μg Se/g diet, so that SELH mRNA levels in Se-deficiency were 8% of levels at 1.0 μg Se/g diet. Plateau breakpoints in pancreas for significantly-regulated transcripts were distributed over a wider range in two groups: 0.02–0.09 μg Se/g diet for MSRB1, SELK, SEPP1, GPX1, SEP15, VIMP, and SEPW1, and 0.14 to 0.21 for SELM, SELU, GPX3, GPX4, and SELH. When all 10 treatments were included in the analysis. TXNRD2 transcript decreased nonsignificantly (*P* = 0.35) in Se deficiency to 24% of plateau levels (data not shown).

### Selenoprotein mRNA Panels

**Fig 9:**
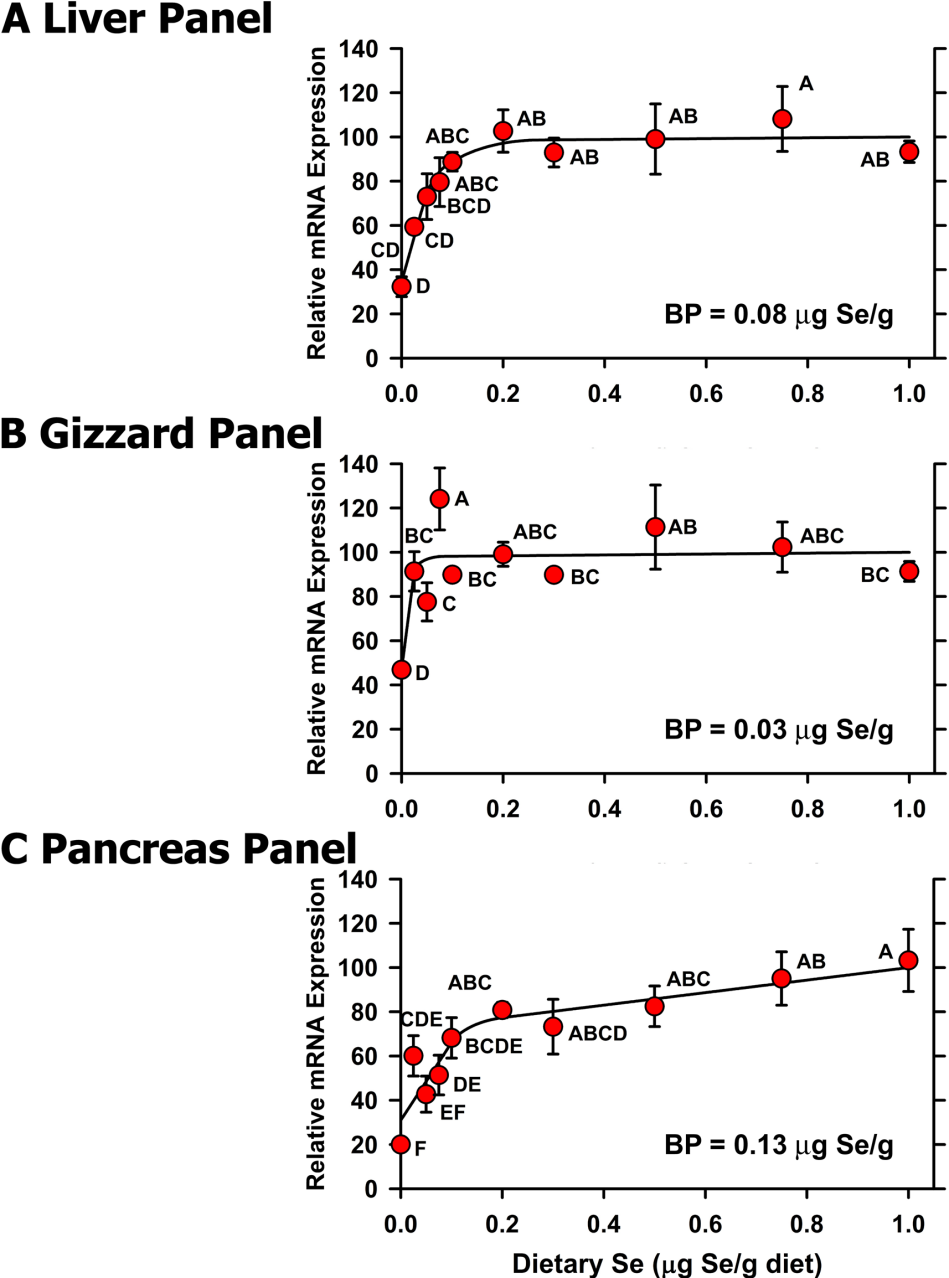
**Effect of dietary Se on transcript panel values in chick liver (A), gizzard (B), and pancreas (C).** Within a tissue, the individual relative transcript levels for each significantly-regulated selenoprotein were averaged to calculate panel values, which were then subjected to the Se response curve analysis as described in the text. Resulting mean±SEM are plotted, and means without a common letter are significantly different (*P*<0.05). Overall level of significance, as determined by ANOVA, is given in Table 3. Se response curve breakpoints (BP) are indicated in each panel, calculated as described in the text.

We previously suggested and showed that panels of selenoprotein transcripts (or molecular biomarkers) could be used to estimate Se status and Se requirements in the rat [30,31]. Thus we averaged the relative expression of the significantly regulated mRNAs in liver, gizzard, and pancreas for each individual chick to calculate a panel value for each bird, which was then used to develop panel Se response curves for liver, gizzard, and pancreas (**Fig 9**). The resulting Se response curves showed clearly defined plateaus for liver and gizzard, where the Se-deficient chicks had composite transcript values that were 32 and 47%, respectively, of Se-adequate plateau levels. Note that the resulting Se-deficient panel means were significantly different from levels in chicks fed 0.075 μg Se/g diet for liver and in chicks fed 0.025 μg Se/g diet for gizzard. In pancreas, the resulting Se response curve increased steeply from 0 to 0.1 μg Se/g diet but then increased at 1/10 the rate after 0.1 μg Se/g diet, such that the Se-deficient level was 26% of the level in chicks fed 0.3 μg Se/g diet, significantly different from levels in chicks fed 0.1 μg Se/g diet, and with a plateau breakpoint at 0.13 μg Se/g diet. While the panel breakpoint in pancreas indicates that the minimum dietary Se requirement is ~0.15 μg Se/g diet, the continued increases in GPX1 and GPX4 activity (Fig 2) and increases in 6 selenoprotein transcripts (Fig 8G–8L) in pancreas beyond 0.15 μg Se/g diet, together indicate that the NRC dietary Se requirement of the chick should be increased to 0.2 μg Se/g diet.

## Discussion

Day-old male chicks in this experiment were fed a basal semi-purified diet containing 22% protein and ~150% of the1994 NRC requirement for vitamins and minerals except Se and vitamin E, and supplemented with 15-times the NRC requirement for vitamin E [1]. With Se supplementation, they grew at >50 g/day over the last two weeks of the study. With or without Se supplementation, there were no gross signs of Se deficiency, including exudative diathesis or pancreatic atrophy [4]. Chicks fed the basal diet without supplemental Se grew at less than half the rate of Se-supplemented chicks; supplementation with 0.025 μg Se/g diet as selenite completely prevented this depression in growth, indicating that the minimum dietary Se requirement for growth of the young male chick under these conditions is 0.025 μg Se/g diet.

We found that both GPX1 and GPX4 enzyme activity are regulated by Se in the chick. As we reported previously for the turkey [20], liver GPX1 and GPX4 enzyme activity levels in Se-adequate birds are distinctly different from levels found in rodents. Se-adequate liver GPX1 and GPX4 activities in chicks are 1/8 and 8-times, respectively, the levels in Se-adequate rat liver [16]. Both liver GPX1 and GPX4 activity are highly down-regulated by Se deficiency in the chick, falling to 2% and 10%, respectively, of Se-adequate levels, whereas in rat liver GPX1 activity is highly regulated but GPX4 activity only falls to ~50% of Se-adequate levels [16]. Chicken gizzard GPX1, gizzard GPX4, liver TXNRD, and plasma GPX3 activities are also all highly down-regulated in Se deficiency, and all have Se-response curve breakpoints in the narrow range of 0.10 to 0.13 μg Se/g diet, supporting a minimum dietary Se requirement for these selenoenzymes of 0.15 μg Se/g diet, the current NRC Se requirement.

Pancreatic atrophy is the disease in the chicken that is prevented by Se but not by modest levels of vitamin E [3,32]. GPX1 and GPX4 activities in chicks fed 0.3 μg Se/g diet are 1/5 and 1/4th, respectively, of the levels in Se-adequate liver, whereas rat pancreas has GPX1 activity of 1/13^th^ of levels in Se-adequate rat liver [33]. Interestingly, but unexplained, pancreatic GPX activity continues to increase after the breakpoint in the chick, in contrast to liver and gizzard, such that chicks fed 1.0 μg Se/g diet have twice the level of GPX1 and GPX4 activity as compared to chicks fed 0.3 μg Se/g diet. Furthermore, GPX1 and GPX4 activities in Se-deficient pancreas fall to only 39 and 25%, respectively, of Se-adequate levels, indicating that in Se deficiency there is either elevated delivery of Se to the pancreas, or increased retention of Se by the pancreas, or both. This provides no explanation as to why the pancreas is the first affected organ in Se deficiency, but it does suggest that homeostatic mechanisms are in place to maintain pancreatic Se levels in the face of Se deficiency in the chick. Protein synthesis rates in the pancreas are higher than for any other tissue [34]; this increased amino acid flux in pancreas may dilute or compete with Se incorporation pathways causing the apparent delays in reaching plateau selenoprotein levels in this tissue. While the minimum dietary Se requirement based on liver and gizzard selenoenzyme activities is 0.15 μg Se/g diet, the higher plateau breakpoints in pancreas strongly suggest that the NRC dietary Se requirement should be raised to 0.2 μg Se/g diet to provide additional protection for the pancreas.

The hierarchy of Se regulation of selenoprotein transcripts in the chick (Table 3) is also distinctly different from the rat [16]. In chick liver, there are 8 transcripts significantly down-regulated with minimum Se requirements ranging from 0.04 to 0.11 μg Se/g, whereas in rat liver, there are 11 down-regulated transcripts in a tight range from 0.03–0.07 μg Se/g. SELH and DIO1 have similar minimum Se requirements in chicks and rats (0.07, and 0.08 in chicks and 0.06 and 0.06 μg Se/g in rats, respectively), and GPX1 transcript requirements are at the top-end of the range in both chick and rat liver. The minimum Se requirement for liver GPX3 transcript in chicks, however, is 0.10 μg Se/g and at the top-end of the range vs. 0.04 μg Se/g in rats, which is at the bottom-end of the range; SEPP1 transcript requirement is similarly near the top of the range in chick liver but at near the bottom of the range in rat liver. These differences offer additional examples of differentially-regulated selenoprotein transcripts that could be used to study the mechanism underlying the sensitivity of some but not all selenoprotein mRNA to Se status [35,36]. Just as observed in the rat [16], the minimum Se requirements for these molecular biomarkers are less than the minimum Se requirements based on the corresponding biochemical biomarkers.

The relative impact of increasing dietary Se on the expression of these biomarkers can be illustrated by plotting relative change in GPX1 and GPX4 enzyme activity and transcripts versus the change in plasma GPX3 activity (S1 Fig). There is a nearly a 1-to-1 correspondence between change in relative liver GPX1 activity versus plasma GPX3 activity over the range of Se-deficient to the 0.3 μg Se/g. Above 0.3 μg Se/g, this relationship breaks-down, as Se is no longer the limiting factor for expression. This figure further shows that liver GPX4 activity increases more steeply than plasma GPX3 or liver GPX1 activity between Se deficient and 0.05 μg Se/g, and that liver GPX4 activity reaches a Se-adequate plateau by 0.075 μg Se/g. This plot also illustrates that GPX1 and GPX4 transcripts reach Se-adequate plateaus at even lower levels of dietary Se, as compared to plasma and liver selenoenzyme activity, as shown in Table 3, Fig 2 and Fig 5. In contrast, the increase in RBC GPX1 activity with increasing dietary Se lags behind the increase in plasma GPX3 activity. This plot shows that the major changes occur between 0.075 and 0.1 μg Se/g for plasma GPX3 and liver GPX1 activity, but for liver GPX4 activity and GPX1 and GPX4 mRNA, the major changes occur between 0 and 0.05 μg Se/g. Clearly plasma GPX3 activity is a good surrogate biomarker for liver GPX1 activity and Se status in the young chick, but RBC GPX1 activity underestimates Se status.

Falls in selenoprotein transcript levels in pancreas in Se deficiency, along with decreases in selenoenzyme activity, further suggest why this organ is targeted by Se deficiency in the chick. SEPP1, SELK, GPX3 and SEP15 mRNAs are expressed in Se-adequate pancreas at levels similar to GAPDH. In Se deficiency, SELK, SELH, SELU, and VIMP mRNA levels all decrease to <20% of Se-adequate levels. Thus declines in these abundant and highly-regulated pancreatic selenoprotein transcripts, and thus pancreas selenoprotein levels, may underlie the targeting of chick pancreas for atrophy in Se deficiency.

In mammals, the Gpx3 gene encodes a N-terminal secretion signal, is highly expressed in rodent kidney, and kidney is the major source of plasma GPX3 in mammals [37]. The chicken GPX3 gene encodes a very homologous signal peptide sequence as well. Thus high expression of GPX3 transcript in gizzard and pancreas suggest that GPX3 secretion by these tissues may play a major role for this gene in the chicken.

The high expression of SEPP1 transcripts in chick liver as well as GPX3 in gizzard (Fig 3) may explain the sigmoidal response curves of GPX1 activity in these tissues (Fig 2). SEPP1 and GPX3 are clearly important secreted transport selenoproteins in mammals [37,38]. Of all the regulated selenoprotein transcripts in liver, SEPP1 mRNA falls the least in Se deficiency, such that in Se deficiency there remains a large pool of SEPP1 mRNA that will direct Se export from liver, resulting in less Se for synthesis of GPX1 in liver, and thus the sigmoidal Se response curve for GPX1 activity in liver. A similar role for GPX3 in gizzard could underlie the sigmoidal Se response curve for GPX1 activity in gizzard.

Plasma SEPP1 protein levels in chicks fed Se-deficient diets for 2 and 4 wk are reported to only fall to 61 and 66%, respectively, of levels in chicks supplemented with 0.3 μg Se/g [39], suggesting that plasma SEPP1 protein may not serve as a good biomarker for Se deficiency in the chick. In humans, the current recommended dietary allowance (RDA) is based on plasma GPX3 activity [40]. Studies conducted by resupplementing Se-deficient adults in China, however, found that more supplemental Se was required to raise plasma SEPP1 protein levels than to raise plasma GPX3 activities to US levels, suggesting that plasma SEPP1 protein would be a better biomarker for determining the RDA [41]. Studies conducted in rats, however, found that plasma SEPP1 protein continues to rise with increasing levels of dietary Se up to 2 μg Se/g [42], showing a lack of a plateau for SEPP1 protein as a Se status biomarker. These studies thus raise the possibility that levels of plasma SEPP1 may reflect dietary Se intake as well as Se status. To better establish the potential of plasma SEPP1 as a Se biomarker, additional studies are needed to assess Se status and requirements by measuring plasma SEPP1 protein in chickens fed graded levels of dietary Se.

One of the objectives of this study was to determine if there were selenoprotein transcripts that could be used as biomarkers for high-Se status. Both the initial screen (3–4 dietary Se supplementation levels) and the full Se response curve analysis (10 Se levels) clearly show that the selenoprotein transcripts in this study are not further increased by high dietary Se levels. Thus at the transcript level, these selenoproteins do not appear to play a role in homeostatic adaptation to supernutritional Se status (≤1.0 μg Se/g).

More than 60 articles have been published studying the impact of Se status and/or form of supplemental Se on selenoprotein transcript expression in chicks and adult chickens. Most of these studies have compared expression in birds fed low-Se diets (0.014–0.03 μg Se/g diet) versus birds supplemented with 0.15–0.3 μg Se/g, but there are no studies that used multiple graded levels of Se spanning the Se-deficient to Se-adequate to supernutritional-Se range. In 2011, Huang et al. [43] studied the expression of 14 selenoprotein transcripts in liver of chicks fed 0.014 μg Se/g diet or supplemented with 0.3 μg Se/g diet. At day 42, EPT1, SEPP1,SEP15, GPX4, and SELS transcripts had the highest expression in liver of Se-supplemented chicks (SELH, GPX3, SELU, SELM, DIO1 were not studied); in Se-deficient chicks at day 42, they reported that 11 of these 14 selenoprotein transcripts (79%) were down-regulated significantly by Se deficiency. Liu et al. [44] reported that SELU, SEPP2, TXNRD1, and GPX4 were highly expressed at day 25 in liver of chicks fed 0.18 μg Se/g diet; in chicks fed the basal diet (0.03 μg Se/g), 16 of the 21 selenoprotein transcripts in that study (76%) were significantly down-regulated, including GPX3, SELU, GPX4, GPX1, SEPP1, and DIO1 (SELH and SELM were not studied). In pancreas, Zhao et al. 2014 [45] reported that SELU, VIMP, MSRB1, and TXNRD1 transcripts were highly expressed in layer chicks fed diets containing 0.23 μg Se/g diet. They further reported that all 25 selenoprotein transcripts (100%) were down-regulated by Se deficiency in chicks fed only 0.03 μg Se/g for 25 days. There are no published studies on chick gizzard but similar results have been reported for other tissues [39,46–53]. Interesting, others have reported that chick liver GPX4 mRNA is decreased by Se supplementation [54], but Huang et al. [39] showed that both chick muscle GPX4 mRNA and protein is increased by Se supplementation. Overall, the relative transcript expression levels in Se-adequate chicks in those studies are similar, with a few exceptions, to levels we found in our study. The number of significantly down-regulated selenoprotein transcripts in those other reports is much greater, however, than in the present study; differences are likely because those studies only included a low-Se and an adequate-Se group, and/or because birds in those studies were not supplemented with higher levels of vitamin E.

The present study was conducted in parallel and at the same time as a study with day-old turkey poults fed virtually the same diets [21]. The growth rate of the Se-supplemented turkeys was half the rate of the chicks in these 4 wk studies, and the minimum Se requirement for growth was 0.05 μg Se/g for turkey poults vs. 0.025 μg Se/g for the chicks. The major difference between these two species was the Se requirement, based on biochemical markers. In turkey poults, the minimum Se requirement based on plasma GPX3 and liver GPX1 activity was 0.3 μg Se/g; using liver GPX4, and gizzard GPX1 and GPX4 activities, the minimum Se requirement was 0.18–0.25 μg Se/g. Thus based on biochemical biomarkers, the minimum Se requirement in the chick is half of that in the poult. The impact of Se deficiency on enzyme activity was almost identical for both species, with the notable exception that pancreas GPX1 activity in the Se deficient turkey poult dropped to 1.2% of Se-adequate levels. There were few major differences in these two species at the transcript level. Highly-expressed transcripts in Se-adequate turkey liver were SEPP1 and GPX4, in turkey gizzard were GPX3 and SEPW1, and in turkey pancreas were GPX1, SEP15, SEPP1, GPX3, SEPW1, and SELK, all similar to highly-expressed transcripts in the chick (Fig 3). The only large distinction was the high level of SEPP2 expression in turkey liver vs. chick liver. Less noticeable, but perhaps germane to the development of disease, levels of GPX1 and GPX4 mRNA, relative to housekeeping gene transcripts, were much higher in all three chick tissues as compared to turkey tissues. The impact of Se deficiency on selenoprotein transcript levels was almost identical for both species, except that the gizzard minimum Se requirements based on GPX1 and GPX4 transcripts and the transcript panel, were about 0.1 μg Se/g higher for the poult versus the chick. These results demonstrate clearly that the chick has a much lower minimum Se requirement than the turkey poult. While Se regulation of transcript levels are similar, there may be key differences between these two species that underlie the targeting of gizzard in the poult vs. pancreas in the chick.

Our initial screen for regulation of transcript expression by Se status illustrates that use of only a limited number of dietary Se levels may identify transcripts that are significantly regulated under these conditions, but that may have limited biological or biomarker impact. When all 10 dietary Se levels (30–50 chicks) were included, the resulting Se response curves show that some of these differences are likely due to biological and experimental variation. The use of multiple graded levels spanning the Se-deficient to Se-adequate range, and inclusion of multiple graded levels on the plateau region, thus better define the response to increasing nutrient in the diet for both biochemical and molecular biomarker response curves (Figs 2 and 5–8). Note how use of multiple graded levels (Fig 6) reveals that some apparent differences are no longer significant, and that these enzyme or transcript levels cannot be used as biomarkers of Se status.

We previously suggested and showed that panels of selenoprotein transcripts (or molecular biomarkers) could be used to estimate Se status and Se requirements in the rat [17,31,32,36]. In the present study, the resulting panel Se response curves (Fig 9) show well-defined plateaus for liver and gizzard, with breakpoints of 0.08 for liver and 0.03 for gizzard; for pancreas, the resulting breakpoint is 0.13 (Table 3). More importantly for each tissue, the Se-deficient value in each panel is significantly and distinctly different from plateau values. This illustrates that use of a panel of Se-regulated transcripts as molecular biomarkers of Se status could be more effective than single individual transcripts to identify Se-deficient animals.

This study used selenite as the supplementary form of Se, in order to directly compare these results with previous studies, and to avoid the complication of organic forms that may be metabolized to selenomethionine and incorporated into general proteins depending on dietary methionine levels [10,55]. A number of older as well as more recent studies have reported that other Se compounds, organic and inorganic, are good sources for dietary supplementation. There is merit in conducting additional studies using these forms of Se with multiple graded Se levels to better characterize the Se response, and using both enzymatic and molecular biomarkers of Se status as well as tissue levels of Se.

In summary, we observed no gross signs of Se-deficiency when diets were supplemented with 15X the NRC vitamin E requirement, but 0.025 μg Se/g was required for optimum growth of male broiler chicks. qPCR for transcripts of all 24 avian selenoproteins found that only 33%, 25% and 50% of selenoprotein mRNAs were down-regulated significantly by Se deficient in liver, gizzard and pancreas, respectively, when 10 graded levels of dietary Se levels were compared; minimum Se requirements for these transcript levels we less than for enzyme activity, just as in rodents, and no selenoprotein transcripts were found to be good biomarkers for supernutritional Se status. Tissue distribution, high expression levels, and Se-regulation of transcripts for the export selenoproteins SEPP1 and GPX3 clearly indicate distinct differences in chicken Se metabolism as compared to mammalian metabolism, which may underlie which tissues are targeted in Se deficiency. Based on GPX1, GPX4, GPX3 and TXNRD activities in liver, gizzard, and plasma, the minimum Se requirement in today’s rapidly growing broiler chick is 0.15 μg Se/g diet; pancreas selenoenzyme activities and transcript levels indicate that the overall Se requirement should be raised to 0.2 μg Se/g diet to provide a margin of safety.

## Supporting Information

S1 Fig Effect of dietary Se on relative change in selenoenzyme activity and transcript level relative to plasma GPX3 activity. To compare the effect of dietary Se on relative change in liver GPX1 and GPX4 activity, liver GPX1 and GPX4 mRNA, and RBC GPX1 activity versus change in plasma GPX3 activity, mean values (from Fig 2 and Fig 5) for each biomarker at each level of dietary Se were expressed as a percent of the mean value at 0.3 μg Se/g diet. The resulting relative expression levels for GPX1 and GPX4 were then plotted versus the relative expression levels for plasma GPX3 activity. Red arrows indicate the μg Se/g level of dietary Se treatment, and Se treatment means are connected by spline curves for each biomarker. The 0.3 μg Se/g diet treatment was selected as 100% as this was the highest minimum dietary Se requirement (Table 3), and because above this level, Se was no longerrate-limiting for expression. The dashed gray line shows a 1:1 unity relationship.
(TIF)
