# Supplementary figures and images for: Selenoprotein Transcript Level and Enzyme Activity as Biomarkers for Selenium Status and Selenium Requirements of Chickens (Gallus gallus)
Source: PLoS One. 2016 Apr 5;11(4):e0152392. doi: 10.1371/journal.pone.0152392 (PMC4821606; doi:10.1371/journal.pone.0152392)

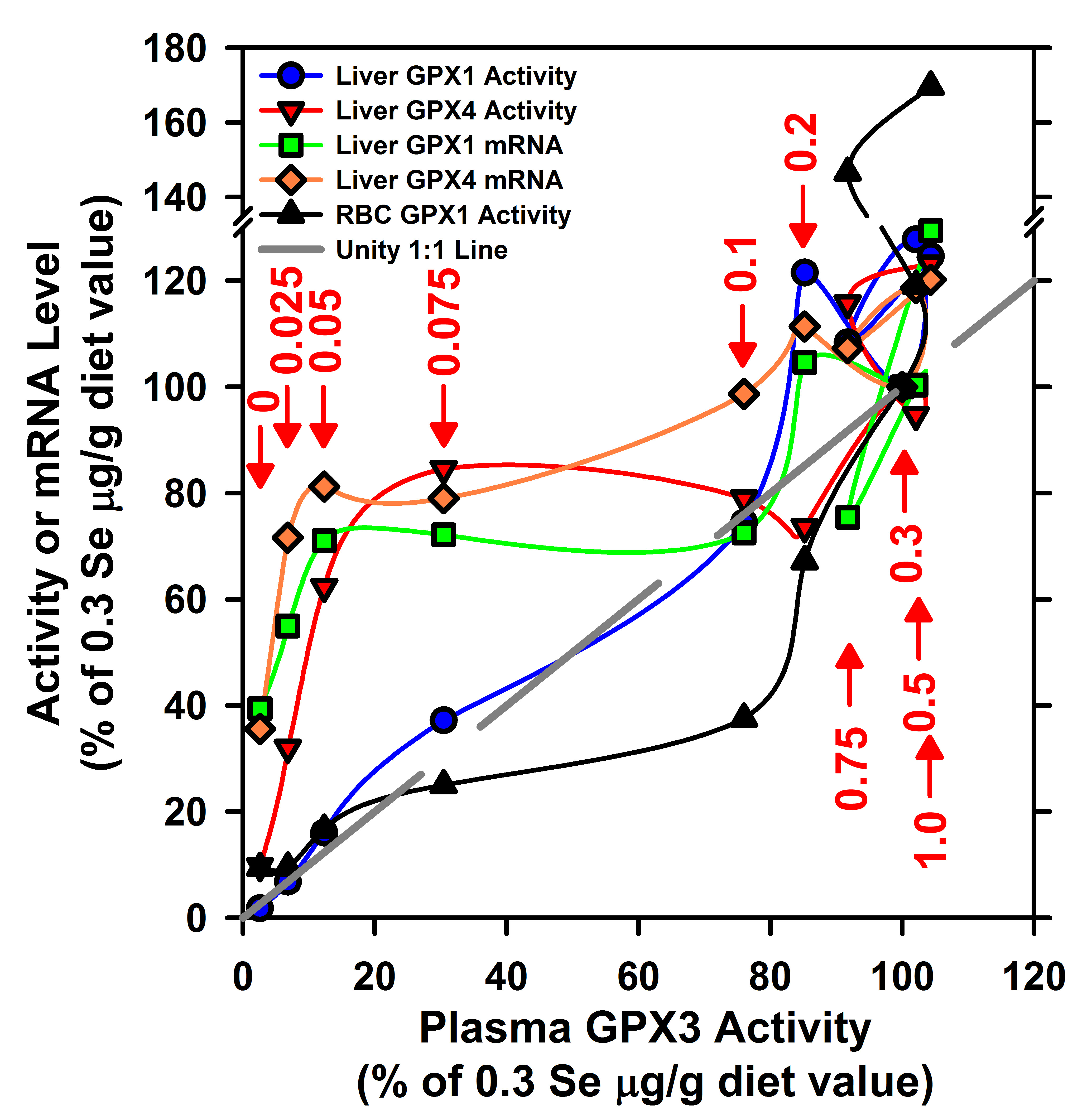

Supplement: S1 Fig — To compare the effect of dietary Se on relative change in liver GPX1 and GPX4 activity, liver GPX1 and GPX4 mRNA, and RBC GPX1 activity versus change in plasma GPX3 activity, mean values (from Fig 2 and Fig 5) for each biomarker at each level of dietary Se were expressed as a percent of the mean value at 0.3 μg Se/g diet. The resulting relative expression levels for GPX1 and GPX4 were then plotted versus the relative expression levels for plasma GPX3 activity. Red arrows indicate the μg Se/g level of dietary Se treatment, and Se treatment means are connected by spline curves for each biomarker. The 0.3 μg Se/g diet treatment was selected as 100% as this was the highest minimum dietary Se requirement (Table 3), and because above this level, Se was no longerrate-limiting for expression. The dashed gray line shows a 1:1 unity relationship. (TIF) [file pone.0152392.s001.tif]
